# Pancreatic β-cell senescence in diabetes: mechanisms, markers and therapies

**DOI:** 10.3389/fendo.2023.1212716

**Published:** 2023-08-31

**Authors:** Jeeyeon Cha, Cristina Aguayo-Mazzucato, Peter J. Thompson

**Affiliations:** ^1^ Division of Diabetes, Endocrinology and Metabolism, Vanderbilt University Medical Center, Nashville, TN, United States; ^2^ Joslin Diabetes Center, Harvard Medical School, Boston, MA, United States; ^3^ Diabetes Research Envisioned and Accomplished in Manitoba Theme, Children’s Hospital Research Institute of Manitoba, Winnipeg, MB, Canada; ^4^ Department of Physiology & Pathophysiology, Rady Faculty of Health Sciences, University of Manitoba, Winnipeg, MB, Canada

**Keywords:** pancreatic beta cells, cellular senescence, type 1 diabetes, type 2 diabetes, monogenic diabetes

## Abstract

Cellular senescence is a response to a wide variety of stressors, including DNA damage, oncogene activation and physiologic aging, and pathologically accelerated senescence contributes to human disease, including diabetes mellitus. Indeed, recent work in this field has demonstrated a role for pancreatic β-cell senescence in the pathogenesis of Type 1 Diabetes, Type 2 Diabetes and monogenic diabetes. Small molecule or genetic targeting of senescent β-cells has shown promise as a novel therapeutic approach for preventing and treating diabetes. Despite these advances, major questions remain around the molecular mechanisms driving senescence in the β-cell, identification of molecular markers that distinguish senescent from non-senescent β-cell subpopulations, and translation of proof-of-concept therapies into novel treatments for diabetes in humans. Here, we summarize the current state of the field of β-cell senescence, highlighting insights from mouse models as well as studies on human islets and β-cells. We identify markers that have been used to detect β-cell senescence to unify future research efforts in this field. We discuss emerging concepts of the natural history of senescence in β-cells, heterogeneity of senescent β-cells subpopulations, role of sex differences in senescent responses, and the consequences of senescence on integrated islet function and microenvironment. As a young and developing field, there remain many open research questions which need to be addressed to move senescence-targeted approaches towards clinical investigation.

## 1 Introduction

Pancreatic islet β-cells are the primary insulin-producing cells in the body, and their cooperation with neighboring endocrine cells in the islets of Langerhans are essential for controlling metabolic responses at a whole organismal level. Malfunction and/or loss of β-cells is a signature of diabetes mellitus, a collection of conditions, such as in Type 1 Diabetes (T1D), Type 2 Diabetes (T2D) and Maturity Onset Diabetes of the Young (MODY), with diverse etiologies commonly marked by hyperglycemia. Recent work from our groups has now established that subpopulations of β-cells undergo senescence during the development and onset of diabetes across mouse models of T1D, T2D and MODY (1–3) and in human pancreas tissue from T1D and T2D donors (1, 2).

Senescence is generally considered to be a terminal stress response that occurs in many different cell types in physiological contexts including embryonic development (4, 5) and aging (6, 7), and in response a wide variety of stressors such as tissue damage (8–10), viral infection (11) and oncogene activation (12). Senescent cells often undergo a DNA damage response leading to cell cycle arrest and progressively acquire resistance to intrinsic apoptosis, chromatin remodeling of gene regulatory networks, metabolic alterations, and development of a complex Senescence Associated Secretory Phenotype, or SASP [reviewed extensively elsewhere (13, 14)]. DNA damage signaling during senescence involves activation of cytoplasmic DNA sensing pathway cyclic GMP-AMP Synthase-STimulator of InterferoN Genes (cGAS-STING), which is also essential for the SASP program (15–17). The SASP secretome consists of a tissue/cell-type specific milieu of growth factors, cytokines, chemokines, proteases, shed receptors and ligands and extracellular vesicles (18) and shows a temporal progression. At early stages SASP is TGF-β-rich and immunosuppressive while at later stages the SASP is highly pro-inflammatory and involves type I interferon signaling (19, 20).The evolution and functions of cellular senescence are complex and have been suggested to demonstrate antagonistic pleiotropy: early in life, senescent cells are beneficial by suppressing cancer and facilitating organogenesis, but paradoxically senescent cells are associated with aging-related disease later in life (21). Although the fundamental points of this theory have been challenged (22) recent evidence has now supported this view (23). The clearance of accumulated senescent cells by the immune system seems to be the primary way senescence is resolved in most tissues during aging and is necessary for tissue homeostasis (24). However, when immune surveillance fails during aging senescent cells accumulate and can impair tissue function and reduce longevity (25) [also reviewed in (26, 27)]. Accumulation of senescent cells, including β-cells, hepatocytes and preadipocytes contributes to the pathology of different forms of diabetes mellitus and metabolic syndrome, and pharmacologic and genetic strategies to mitigate senescence have shown beneficial effects in mouse models (1–3, 28–30), underscoring the pathogenic effects of senescent cells in these diseases.

**Figure 1:**
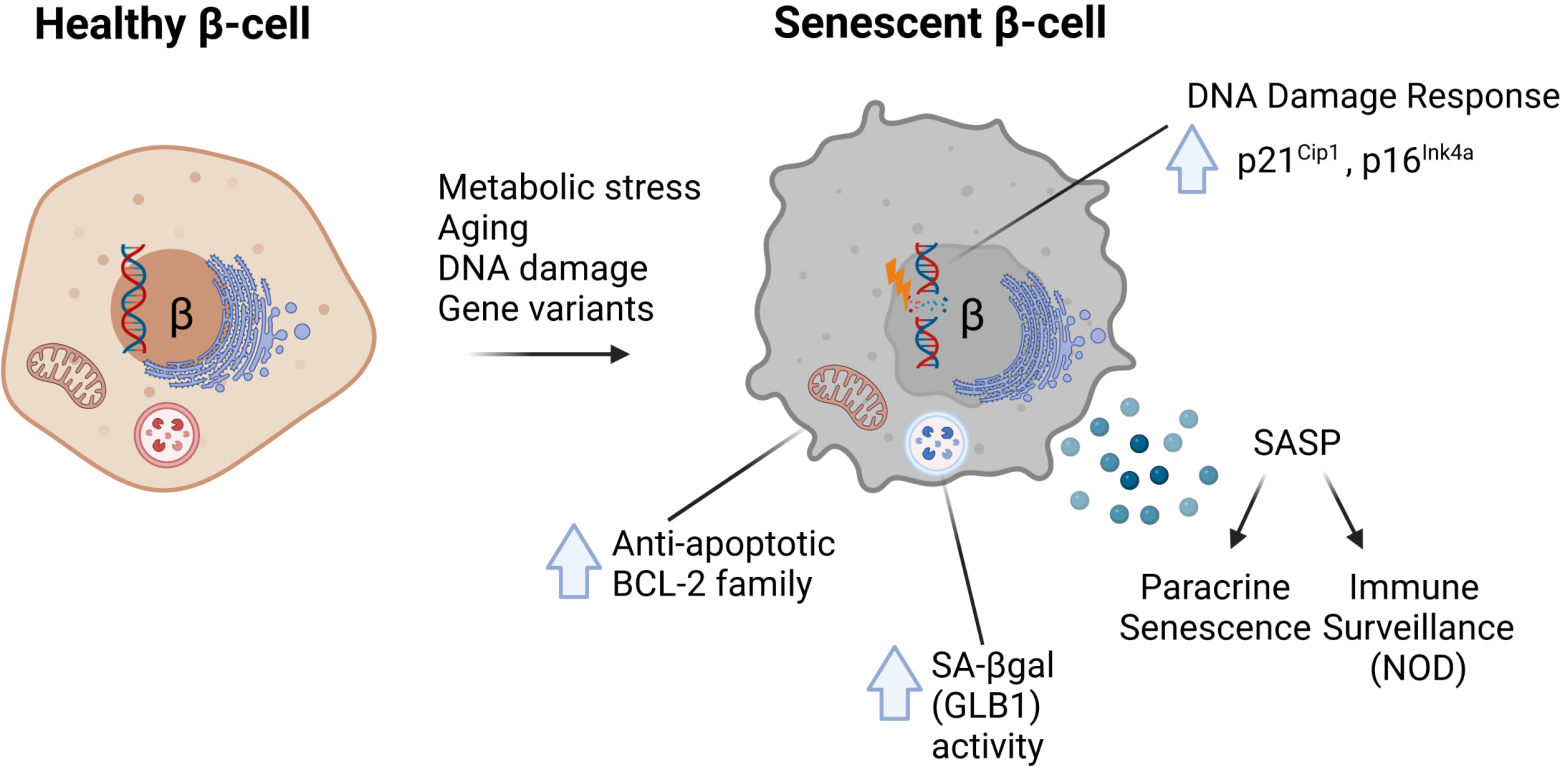
Common molecular features of senescent β-cells characterized in mouse models of diabetes. In response to metabolic stress, aging, direct DNA damage or the MafA*^S64F/+^* gene variant, β-cells can develop senescence defined by several common features. These include (1) increased DNA damage and upregulation of Cdk inhibitors p21^Cip1^ and/or p16^Ink4a^; (2) increased activity of lysosomal enzyme GLB1, also referred to as senescence-associated βgalactosidase (SA-βgal); (3) increased expression of anti-apoptotic BCL-2 family proteins which mediate a prosurvival state; and (4) development of a Senescence-Associated Secretory Phenotype (SASP).

The molecular signatures associated with senescence vary by cell type and context but recent work has established at least four common hallmarks of β-cell senescence across different models (1–3) (**Figure 1**). These include: [1] increased markers of DNA damage and upregulation of cyclin-dependent kinase inhibitors p21^Cip1^ and/or p16^Ink4a^; [2] increased activity of the lysosomal enzyme GLB1 also called senescence-associated βgalactosidase (SA-βgal); [3] activation of a prosurvival pathway, such as the anti-apoptotic BCL-2 family of proteins; and [4] the development of a functional SASP (each discussed in depth below). Moreover, senescent β-cells maintain insulin expression and do not seem to acquire expression of other islet hormones. Together these features and molecular markers clearly distinguish senescence from other stress responses in the β-cell, such as apoptosis or altered β-cell identity (31). Additional molecular markers of senescence vary by disease model and context; however, all of these common features are readily assayed at the gene expression, protein and cellular/phenotypic levels (**Table 1**). Despite these advances, our knowledge in this emerging area stems from a relatively small number of studies and thus there is much to be learned about the molecular mechanisms of senescence in the β-cell, heterogeneity of senescent signatures depending on context, and how specific signaling pathways in senescent β-cells may be exploited to develop therapies for diabetes subtypes. In this review, we summarize the current state of knowledge of β-cell senescence in T1D, T2D and MODY, and propose a set of best-practices for the field for evaluating β-cell senescence to help guide its potential for clinical translation.

**Table 1:** Published markers of β-cell senescence in mouse models of diabetes and human cells/samples.

Diabetes Models/Samples	Senescence Feature	Trait measured	Gene	Protein	Expected change	Detection Assays	Species	Citations	Comments
**Mouse**: C57BL6 HFD Insulin Resistance, Aging; C57BL6 MafA^S64F^ males; NOD/ShiLtJ female mice **Human**: T2D donor islets and pancreas; EndoC-βH2 cells expressing MAFA^S64F^; T1D donor pancreas, direct DNA damage to normoglycemic donor islets, EndoC-βH5 cells	Cell cycle arrest	Activation of p16-pRB axis	*CDKN2A/Cdkn2a*	p16^INK4A^	Up	qPCR, IF, RNASeq	Human, Mouse	(1, 2, 32, 33)	Note 1
		Activation of p53-p21 axis	*CDKN1A/Cdkn1a*	p21^CIP1^	Up	qPCR, IF, RNA-seq, Western blot, Flow Cyto, scRNASeq	Human, Mouse	(1–3, 32–36)	Note 2
			n/a	ATM (Ser1981-P)	Up	Western blot	Human	(34)	
	Lysosomal compartment	Increased lysosomal compartment and activity	*GLB1, Glb1*	SA-βgal	Up	Xgal or fluorescence staining	Human, mouse	(1–3)	Note 3
	Prosurvival phenotype	Anti-apoptotic BCL-2 family members	*BCL2L1; Bcl2a1d*	Bcl-2, Bcl-xL	Up	IF, qPCR, RNA-seq	Human, mouse	(1, 3, 34)	Note 4
	Nuclear reorganization	Chromatin changes	*n/a*	HMGB1	Nuclear exclusion	IF	Human, mouse and MIN6 cells	(32, 33, unpublished)	Note 5
		DNA damage foci	*n/a*	53BP1	Up	IF	Human, mouse	(2, 3)	
			n/a	gamma-H2A.X	Up	IF, Western blot	Human, mouse	(1, 3, 34)	Note 6
		Nuclear integrity, gene expression	*LMNB1*	LMNB1	Down	qPCR, IF	Human, mouse	(3)	
	Senescence associated secretory phenotype (SASP)	SASP gene expression and secretion	*Il6*	n/a	Up	qPCR	Mouse	(2)	
			*IL1A/Il1a*	n/a	Up	qPCR	Human, Mouse	(2)	
			*Ctsb*	n/a	Up	qPCR	Mouse	(2)	
			*Plau*	n/a	Up	qPCR	Mouse	(2)	
			*Cd68*	n/a	Up	qPCR	Mouse	(2)	
			*Serpine1*	n/a	Up	qPCR	Mouse	(2)	
			*Igfbp3*	n/a	Up	qPCR	Mouse	(2)	
			*Igfbp5*	n/a	Up	qPCR	Mouse	(2)	
			*Fgf2*	n/a	Up	qPCR	Mouse	(2)	
			*Cxcl2*	n/a	Up	qPCR	Mouse	(2)	
			*Cxcr4*	n/a	Up	qPCR	Mouse	(2)	
			*Lamb1*	n/a	Up	qPCR	Mouse	(2)	
			*Lamc1*	n/a	Up	qPCR	Mouse	(2)	
			*Ccl2*	n/a	Up	qPCR	Mouse	(2)	
			*TNFa*	n/a	Up	qPCR	Mouse	(2)	
			*CCL3*	n/a	Up	qPCR	Human	(2)	
			*CCL4*	n/a	Up	qPCR	Human	(2)	
			*CCL5*	n/a	Up	qPCR	Human	(2)	
			*CXCL1*	CXCL1	Up	qPCR, Luminex, RNAseq	Human	(2)	
			*CXCL10*	n/a	Up	qPCR	Human	(2)	
			*TNFA*	n/a	Up	qPCR	Human	(2)	
			*IL6*	IL-6	Up	qPCR, Luminex, IF	Human, Mouse	(1, 2, 32, 35)	
			*ICAM3*	n/a	Up	qPCR	Human	(3)	
			*IGFBP2*	n/a	Up	qPCR	Human	(3)	
			*n/a*	IGFP3	Up	Luminex	Mouse	(1, 35)	
			*IGFBP4*	IGFBP4	Up	qPCR, Luminex, RNAseq	Human	(3, 34, 35)	
			*Ankrd1*	n/a	Up	qPCR	Mouse	(3)	
			*Cxcl10*	n/a	Up	qPCR	Mouse	(35)	
			*Igfbp4*	n/a	Up	qPCR	Mouse	(35)	
			*IL6*	IL-6	Up	qPCR, IF	Human	(1, 32, 34, 35)	
			*Flnb*	FLNB	Up	IF, qPCR	Mouse	(1, 34)	
			*Mmp2*	MMP2	Up	qPCR, Luminex, IF	Mouse	(1, 34, 35)	
			CXCL8	IL-8	Up	qPCR, Luminex	Human	(34, 35)	
			*n/a*	SERPINE1	Up	Luminex	Mouse	(1)	
			*n/a*	MMP3	Up	Luminex	Mouse	(35)	
			*n/a*	MMP12	Up	Luminex	Mouse	(35)	
			*GDF15*	GDF15	Up	Luminex, RNA-seq	Human	(34)	
			*TNFRSF10C*	TNFRSF10C	Up	Luminex, RNA-seq	Human	(34)	
			*SERPINA6*	n/a	Up	RNA-seq	Human	(34)	
			n/a	Various	Up	Proteomic analysis. Aptamer based	Mouse, human, MIN6	(33)	Note 7

1. Low expression levels, very low Ct values. Expression is unchanged at protein level in T1D donors. Expression is unchanged at mRNA level in human donor islets following DNA damage-induced senescence. Poor antibody specificity is a common issue in mouse immunostaining.

2. Nuclear localized in T1D donor β-cells, increased in human donor islet/beta cells and EndoC cells following DNA damage-induced senescence. High cytoplasmic stain in T2D and Insulin resistance mouse models.

3. β-cells have high endogenous β-gal activity, therefore incubation times need to be optimized.

4. Bcl-2 is required for prosurvival state of senescent β-cells in NOD mice. BCL-XL is upregulated at mRNA level during DNA damage senescence in EndoC-βH5 cells and human islets. BCL-2 was upregulated in MAFA^S64F^-expressing EndoC-βH2 cells. Whether BCL-2 or BCL-XL each control the prosurvival phenotype in senescent human β-cells was not determined from these studies.

5. Reliable and reproducible staining of both dispersed cells and pancreas sections.

6. High background is a common issue.

7. Unable to determine absolute concentrations from this assay.

n/a, Not Applicable.

## 2 Strategies to evaluate β-cell senescence

A universal senescence signature that defines all senescent cells across tissues and aging has not been identified (37), underscoring the complexity of this cellular state. Therefore, it is critical to avoid ambiguity and make an accurate determination of whether senescence is involved in particular model. It is recommended that several independent markers representing distinct features of senescence are tested in parallel to establish the presence of senescent β-cells. As discussed earlier, senescence is inherently heterogeneous, and markers of β-cell senescence are likely to vary based on species (mouse vs. human), stressors involved, disease state, and cellular environment (*in vitro* versus *in vivo*). Published approaches which have been used to identify mouse and human β-cell senescence are summarized in **Table 1**. In this section we briefly discuss approaches for assaying these features and their utility for characterizing senescent β-cells in mice and humans.

### 2.1 SA-βgal activity

The lysosomal SA-βgal enzyme activity levels increase dramatically during senescence in many cell types and can be detected using X-gal staining (38) or C_12_FDG fluorescence staining (39). Mouse and human β-cells with high SA-βgal activity accumulate during aging and exhibit other features of senescence (2, 40) (**Table 1**), making this a useful approach for scoring or sorting presumed senescent β-cells. However, it should be kept in mind that lysosomal SA-βgal activity is not required for senescence (41) nor it is specific to senescence, as it can occur during other cellular responses such as autophagy or with high cell culture confluency (42, 43). Therefore, as a stand-alone marker, it is insufficient to prove that a β-cell is senescent, and additional molecular testing is recommended.

### 2.2 Expression of Cdk inhibitors

Senescent β-cells in both mouse and human frequently upregulate p21^Cip1^ and/or p16^Ink4a^ and both should be monitored at the mRNA and protein levels. Validated primer sets are available to detect mRNA levels of *Cdkn1a* (encoding p21^Cip1^) or *Cdkn2a* (specific to exons encoding p16^ink4a^). Specific and validated antibodies for p21^Cip1^ are commercially available, however, available antibodies to detect p16^Ink4a^ in mouse samples are often nonspecific [e.g. its detection in non-senescent macrophages (43)], and claims of specificity must be empirically established using appropriate controls. Also, care should be taken to note the localization patterns of each of these proteins in immunohistochemistry studies, as cytoplasmic localization for either one could indicate non-canonical functions. Furthermore, results in immortalized cell culture models (i.e. MIN6, HeLa) should be carefully interpreted as these cells have dysregulation of cell cycle regulators at baseline.

### 2.3 Loss of nuclear LaminB1 and HMGB1

Another common feature of senescence across different cell types is loss of nuclear integrity and reduced expression of nuclear LaminB1 (44) and HMGB1 (45). Loss of nuclear integrity impacts transcriptional activity as LaminB1 and HMGB1 proteins dynamically interact with active versus repressive chromatin to establish distinct topological domains, thus influencing the production of SASP (46, 47). Loss of LaminB1 and HMGB1 alters the spatial repositioning of heterochromatin to facilitate the formation of senescence-associated heterochromatin foci (SAHF) (reviewed in (48). Detection and localization of these markers can be assessed using immunocytochemistry or immunohistochemistry (**Table 1**). While loss of LaminB1 and HMGB1 have been found in some contexts of β-cell senescence in mice and cultured human β-cells (3, 32, 33), it is currently unclear whether this occurs across all models in which senescent β-cells are present and whether it occurs in resident senescent human β-cells in clinical disease.

### 2.4 Markers of DNA damage response

Markers of DNA damage are often seen in senescent cells, particularly with DNA damage-induced senescence (49). Enriched, punctate nuclear staining Trp53 binding protein 1 (53BP1) and phosphorylated H2AX (gH2AX) by immunostaining protocols mark double stranded DNA breaks implicated in driving cellular senescence (reviewed in (50). Lack of specificity of commercially available gH2AX antibodies have been noted across islet studies and therefore appropriate controls should always be included and results interpretated with caution.

### 2.5 SASP

The culture medium conditioned by senescent cells is enriched with secreted proteins which can promote the transformation of premalignant cells or induce phenotypic changes in neighboring cells, including paracrine senescence (51, 52) or tissue regeneration (10). Such enhanced secretory activity can also activate the adaptive Unfolded Protein Response (UPR) (19) although whether this occurs in β-cell senescence requires investigation. β-cell SASP repertoire consist of a variety of secreted molecules, including cytokines, chemokines, matrix metalloproteases, and growth factors (1–3, 33, 34), and those factors secreted from senescent β-cells across diabetes contexts have been identified using candidate and discovery approaches (**Table 1**) and show considerable differences between mouse and human β-cells (33, 34). SASP can be monitored at multiple levels, including mRNA, protein expression and secretion. To establish that senescent β-cells exhibit SASP, the SASP factors in question should, at the least, be monitored at the secretory level, as this is the only way to confirm that the factor is part of their secretome. In models involving the immune system, care should be taken to rule out contributions from immune cells, since some immune cell types secrete factors that overlap with common SASP constituents, such as IL-6 and IL-8. A *bona fide* SASP also should possess biological activity, which is best demonstrated by paracrine assays using the conditioned media from islets containing senescent β-cells and culturing this media with either naïve islets/cell lines or with immune cells (1, 2). A biologically relevant SASP can be confirmed by paracrine assays, demonstrating that the senescent β-cell conditioned media induces phenotypic changes in the naïve cells, such as paracrine senescence (1–3), or chemotaxis from immune cells in culture (1).

### 2.6 Prosurvival phenotype

Senescent cells activate anti-apoptotic pathways to resist normal programmed death signals [reviewed in (27)]. Senescent β-cells in mouse models of diabetes have thus far been shown to upregulate anti-apoptotic members of the BCL-2 family (1–3), although other pathways may also contribute. Key family members include Bcl-2, Bcl-xL, Bcl-w and Mcl-1 (1–3). As has been found in other senescent cell types, these changes can be assayed at the mRNA and protein levels (53). To confirm that the upregulation of one or more of these factors confers a prosurvival phenotype, small molecule BH3 mimetic compounds (such as ABT-263, ABT-737 and/or ABT-199) often used in cancer therapy (reviewed in (54) and which have been shown to possess senolytic activity (1, 53, 55), can be used to inhibit these proteins. Senescent cells are preferentially induced to undergo apoptosis in the presence of these compounds if the prosurvival mechanism employs anti-apoptotic BCL-2 family proteins (53, 55). It should be noted that there are a variety of other prosurvival mechanisms co-opted by senescent cells (56, 57), and these alternative pathways can also be explored if an upregulation of BCL-2 family members is not detected.

## 3 Molecular mechanisms of β-cell senescence

While cell cycle arrest can be mediated by several proteins in the Cyclin-dependent kinase (Cdk) inhibitor family, p21^Cip1^ and p16^Ink4a^ have emerged as the major drivers of senescence in a broad range of cell types. This conclusion was established from key early studies on human fibroblast senescence models, genetic knockout mice and cancer (58–61) and has been extensively confirmed in subsequent studies [reviewed in (62)]. Activation of p21^Cip1^ (encoded by *Cdkn1a*) and p16^Ink4a^ (an alternative splice product encoded by the *Cdkn2a* locus) tumor suppressor pathways are often upregulated by different cellular stressors, including (but not limited to): oncogene activation, telomere shortening, DNA damage, protein aggregation and reactive oxygen species (ROS) [reviewed in (13)]. This section will address the role of p21^Cip1^ and p16^Ink4a^ in β-cells in the context of function, proliferation and senescence.

### 3.1 p21^Cip1^ in β-cell proliferation and function

*In vitro*, induction of DNA damage leads to stable p21^Cip1^ expression in mouse β-cell lines MIN6 and NIT-1 concomitant with a senescent-like growth arrest (2, 33, 34). Inhibition of p21^Cip1^ during acute DNA damage in MIN6 cells led to apoptosis (63), suggesting a key role of this Cdk inhibitor in promoting survival of β-cells following DNA damage. Given their role as cell-cycle inhibitors, several reports studying p21^Cip1^ and p16^Ink4a^ first elucidated their roles as inhibitors of β-cell proliferation (64, 65). Interestingly, in a model of constitutively active Akt, mouse β-cells showed increased β-cell mass and proliferation via cyclin D1 and cyclin D2 with increases of p21^Cip1^ and p16^Ink4a^ (66), however, another study demonstrates that mice with reduced p21^Cip1^ in the setting of constitutively active Akt signaling showed fasting and fed hypoglycemia, hyperinsulinemia and improved glucose tolerance (65). In contrast, opposite effects of p21^Cip1^ in β-cell function were observed in mice deficient in the cell cycle regulator Sei1. Sei1 promotes proliferation through the assembly of Cdk4-cyclin D and transcription factor E2F1, and mice deficient in this regulator with increased p21^Cip1^ are glucose intolerant with impaired insulin secretion and reduced β-cell mass (67). These findings suggests that the molecular context in which p21^Cip1^ is increased affects β-cell responses.

The subcellular localization of p21^Cip1^ also plays an important role in its regulation of β-cell proliferation. Using INS-1E cells with increased expression of protein kinase PKCδ, it was shown that p21^Cip1^ was excluded from the nucleus favoring its cytosolic localization, which led to increased proliferation (64). Other work has shown an anti-apoptotic role for cytoplasmic p21^Cip1^ independent of its cell cycle inhibitor activities (68). Additional experiments to understand the role of cytosolic versus nuclear p21^Cip1^ in models of β-cell senescence will be important since there are studies that report both its nuclear localization (1, 32) in models of T1D and cytoplasmic localization (2, 69) in models of T2D. Unfortunately, none of these studies addressed β-cell senescence, and *Cdkn1a* knockout (KO) mice show normal islet mass, β-cell replication rates, and function (70). Therefore, further studies are required in models of β-cell-specific deletion and overexpression that focus on the roles of these regulators in terms of spatial localization, temporality, and duration of expression.

### 3.2 p16^Ink4a^ in β-cell proliferation and function

p16^Ink4a^ was also identified as an inhibitor of β-cell proliferation through its action on kinases Cdk4 and Cdk6, which are critical for β-cell proliferation in both mouse and humans, respectively (71, 72). Importantly, a second tumor suppressor is encoded at the *Cdkn2a* locus (p19^Arf^ in mouse, p14^Arf^ in humans) which can also play a role in cell cycle inhibition by altering the Mdm2/p53 axis (62). Most studies have focused on p16^Ink4a^ and have not selectively altered expression of p19^Arf^ without an impact on p16^Ink4a^. Therefore, comparatively less is known about specific functions of p19/p14^Arf^ in β-cells. Here we focus on the roles of p16^Ink4a^ in β-cell senescence.

Several studies have demonstrated that expression of p16^Ink4a^ at the protein and mRNA levels across tissues increases with age in humans and mice (1, 2, 40, 69, 73, 74). In mice, this age-dependent increase in expression was correlated with decreased methylation of the *Cdkn2a* gene locus (74) and reduced repressive histone modifications including Histone H3 Lys 27 trimethylation (H3K27me3) and Histone H2B Lys119 ubiquitination (H2BK119ub) (75, 76). Expression of p16^Ink4a^ in human β-cells is also age-dependent: its expression was detected in 8% of prenatal β-cells and increased to about 63% of adult β-cells (77), however, absolute quantities of p16^Ink4a^ protein need to be interpreted with care due to the challenges in the field with specificity of p16^Ink4a^ antibodies, mostly in mouse, to detect it by immunohistochemistry (50).

The age-related increase of p16^Ink4a^ in pancreatic β-cells has been suggested to be responsible for the age-related limited regeneration of this cell type (73). Supporting this concept, mice that overexpress p16^Ink4a^ had decreased islet proliferation while proliferation was increased in aged p16^Ink4a^-deficient mice (73). The regulation of β-cell proliferation by p16^Ink4a^ is dependent on PTEN status, as p16^Ink4a^ was downregulated when PTEN was lost as a result of cyclin D1 induction and activation of E2F transcription factors (78). In turn, activation of E2F transcriptional-factors led to methylation of *Cdkn2a* promoter, thus inhibiting its expression. Furthermore, a study using an overexpression model of p16^Ink4a^ driven by the *Ins2* promoter has shown that β-cell function as assessed by glucose stimulated insulin secretion (GSIS) was improved while it was decreased in islets from p16^Ink4a^-deficient mice (40), suggesting that this Cdk-inhibitor is also important for β-cell function by inducing β-cell maturation.

However, other studies suggest that p16^Ink4a^ expression has a deleterious effect on insulin secretion. Mice with telomerase haploinsufficiency with increased p16^Ink4a^ expression in β-cells were insulin intolerant suggesting a primary defect on β-cell function (79). A knockdown of *CDKN2A* in the EndoC-βH1 human β-cell line increased insulin secretion (80). In fact, several noncoding genetic signals in the *CDKN2A/B* locus associate with high risk for human T2D (81) and loss of β-cell function (80) by GWAS analysis. At least four independent signals have been described at this locus (rs7856455, rs10757282, rs10811661, and rs1575972) (82). In particular, the risk allele (T) of the rs10811661 SNP correlates with reduced insulin secretory capacity (83, 84), higher HbA1c (p=2.15-10), prevalence of T2D and higher transcription of *CDKN2A* transcript in the human pancreas (while the other SNPs are not). In human studies, individuals from familial melanoma kindreds with a heterozygous loss of function of *CDKN2A* had increased insulin secretion supporting a deleterious effect of this Cdk-inhibitor on human β-cell function (80).

While these studies have advanced our understanding of the roles of p21^Cip1^ and p16^Ink4a^ in β-cells, studies addressing their distinct roles in physiologic β-cell senescence (and functional maturity) versus pathologic senescence in disease remain open. Sequential activation of p21^Cip1^ and p16^Ink4a^ during senescence progression has been recently reported in primary human fibroblasts in response to three well-established senescence-inducing stimuli: replication, oncogene-induction and irradiation (20). Under these conditions, cellular senescence proceeded through an early DNA damage-response phase characterized by p21^Cip1^ upregulation followed by a SASP response which coincided with an increase in p16^Ink4a^ (20). Indeed, in β-cells from adult mice treated for two weeks with insulin receptor antagonist S961 modeling insulin resistance, expression of *Cdkn1a* increased transiently followed by a peak of *Cdkn2a* expression (33). Interestingly, this duration of S961 treatment also induces hyperinsulinemia in the setting of hyperglycemia with proliferation of a subset of β-cells (85). scRNASeq of islet cells from S961-treated mice suggest distinct subpopulation of proliferative and senescent β-cells (33). Whether the proportion of β-cells entering these cell fates (i.e. proliferation and senescence) depends on the dose and/or duration of S961 exposure remains to be discerned. In contrast, in T1D donor pancreas tissue and in a human islet model of DNA damage-induced senescence, *CDKN1A* and p21^Cip1^ upregulation occurs without any increase in *CDKN2A* or p16^Ink4a^ (1, 34, 35), suggesting that the response of p21^Cip1^ and p16^Ink4a^ in β-cells depends on the stressor. In some models, these results support a temporal progression of β-cell senescence during metabolic stress in which *Cdkn1a* is expressed in early stages, whereas *Cdkn2a* persists as a marker of established cellular senescence (33). Additional work has shown that the senescence growth arrest in fibroblasts enforced by p16^Ink4a^ and p21^Cip1^ can be decoupled from SASP (86), the latter of which being driven by other mechanisms occurring during senescence, such as persistent DNA damage response signaling (49), chromatin remodelling (87, 88) and activation of the p38/MAPK pathway (89). Whether this is also true in the context of senescence in β-cells remains to be determined. Overall, questions remain regarding the circumstances in which p21^Cip1^ and p16^Ink4a^ are upregulated with respect to the other, whether their activation is sequential or occur in parallel and whether they can enforce different forms of senescence to drive heterogeneous senescent β-cell populations.

### 3.3 Heterogeneity in β-cell senescence: Do p21^Cip1^ and p16^Ink4a^ mark different subpopulations of senescent β-cells?

Senescence is being recognized as a highly heterogeneous phenomenon, both *in vitro* (90, 91) and *in vivo* (14, 37), and there is accumulating evidence for distinctions between senescent cell populations expressing p21^Cip1^ versus p16^Ink4a^. Whereas early work viewed p16^Ink4a^ as a near-universal marker for senescent cells *in vivo* and developed a suite of molecular and genetic tools based around the detection and ablation of p16^Ink4a+^ cells, a shift in the field has occurred in recent years to focus on p21^Cip1^-expressing senescent cells. Notably, a recent study showed that p21^Cip1^ and p16 ^Ink4a^ expression elicit differences in SASP, where the p21^Cip1^- but not p16 ^Ink4a^ -driven SASP is sufficient to trigger immune surveillance and removal of senescent cells *in vivo* (92). Similarly, selective deletion of p21^High^ cells in adipose tissue improves metabolic health in the HFD mouse model (93) and depletion of p21^Cip1+^ but not p16^Ink4a+^ senescent cells improves bone health following radiation exposure (94). In contrast with early work showing benefits of ablating p16^Ink4a+^ cells on healthspan in mice, a recent study showed that p16^High^ cells are critical for healthy aging (95), thus challenging the notion that all p16^Ink4a^-expressing senescent cells are deleterious.

Evidence for heterogeneity of senescent β-cell subpopulations expressing predominantly p21^Cip1^ or p16^Ink4a^ is apparent in mouse and human models relevant to T1D. In the NOD mouse model, the percentage of β-cells expressing p21^Cip1^ is around 5-10% by 14-15 weeks of age, whereas the percentage expressing p16^Ink4a^ is ~30-40% (1). Indeed, the frequency of p21^Cip1^-expressing β-cells in late-stage euglycemic NOD mice most closely matches that of β-cells expressing other later senescence markers, such as Bcl-2 and SASP factors Mmp2, IL-6 and Igfbp3. While both p21^Cip1^ and p16^Ink4a^-expressing β-cell subpopulations are sensitive to BH3-only mimetic inhibitors with senolytic activity [i.e. ABT-737 and ABT-199, which mimic binding of the BH3-only initiator proteins to anti-apoptotic members (54)], the differences in the depletion of these populations is notable. When islets isolated from NOD mice at 14 weeks of age are cultured in the presence of senolytic ABT-737, nearly all the p21^Cip1^ expression is depleted, whereas only about 50% of the islet p16^Ink4a^ expression is lost (1). In contrast, in aged human donor islets, senolytic compound ABT-263 mainly depletes *CDKN2A* expression (p16^Ink4a^ mRNA) rather than *CDKN1A* (2). In human T1D donor pancreas, the subset of p21^Cip1+^ β-cells is significantly higher than in healthy controls with autoantibody-positive donors (1 or 2 AAbs) and in contrast, p16^Ink4a+^ β-cells were no different among the donor groups (1). p16^Ink4a^ expression was already quite high even in the youngest pediatric donors examined in that study (aged 12-14), consistent with the age-related β-cell maturation and cell cycle exit. Replicating this observation in a culture model, DNA damage-induced senescence in islets from healthy donors leads to stable activation of p21^Cip1^ but not p16^Ink4a^ (1, 34, 35) and more recently p21^Cip1^ expression was confirmed in β-cells in this model (36). Additional evidence for distinctions between p21^Cip1^ and p16^Ink4a^ in senescent β-cells was recently shown with the *INK-ATTAC* mouse model, which revealed that ablation of p16^Ink4a^-expressing β-cells left a remaining senescent β-cell population expressing SASP factor genes (96). In future work, it will be important to investigate the molecular features that distinguish p16^Ink4a^ -expressing β-cells from p21^Cip1^-expressing β-cells in the context of T1D and T2D mouse models and human islets.

## 4 Lessons on β-cell senescence from genetic models

In studying the molecular programs and effectors of senescence, much can be learned from the relationships with apoptosis. Senescence and apoptosis have been proposed as complementary cell fates (97), where senescence is often favored when the stress or damage is irremediable but not as severe or extensive as compared with apoptosis (98). Yet how these distinct stress responses in β-cells relate to one another during the development and progression of diabetes is poorly understood. In mouse models for T1D (e.g. NOD mouse) and T2D (e.g. HFD mouse), only a minority of β-cells develop senescence whereas others undergo apoptosis or (mal)adaptive stress responses. Thus, it is important to consider that β-cell senescence in the context of diabetes does not occur in isolation from other stress responses such as apoptosis.

Studies from whole-body mouse genetic models provide instructive examples of the intricate balance between senescence and apoptosis in β-cells. Mutations altering the p53 pathway or mutations affecting β-cell replication and DNA repair result in a heterogeneous population of β-cells, where some undergo senescence and others apoptosis (99–101). Notably, α-cells, though harboring the same mutation are not generally affected in these models (**Table 2**). For instance, mice harboring knock-out of the DNA ligase *Lig4* (leading to increased endogenous unrepaired DNA strand breaks) and a point mutation in *Tp53* (R172P) that mitigates p53-induced apoptosis, leads to very early onset of diabetes, involving both senescent β-cells and progressive loss of β-cell mass due to p53-independent apoptosis (100). In line with these findings, selective ablation of *Tp53* failed to ameliorate dysglycemia induced by genetic modifications, diet, or pharmacologic agents (103), however β-cell senescence was not addressed. Similarly, deletion of the chromosome licensing factor *Pttg1* (encoding the protein Securin), leads to a mixture of both senescent and apoptotic β-cells (99). In both the p53^R172P^;*Lig4* ^-/-^ mutants and the *Pttg1^-/-^* mutants, senescent and apoptotic β-cells were present, and progressive loss of β-cell mass and diabetes occurred at relatively young ages (5-10 weeks). Furthermore, mice harboring a whole-body point mutation that reduces expression of the DNA repair gene *Ercc1*, show accelerated aging, glucose intolerance and reduced β-cell mass with the accumulation of β-cells expressing senescence markers (101). *Ercc1* hypomorphic mutant mice also exhibit senescent cell accumulation in other organs which may contribute to the metabolic dysfunction observed, nevertheless, this study affirms the importance of efficient repair of endogenous DNA damage for β-cell health. What causes some β-cells to activate a senescence program and others to undergo apoptosis in the same islets, in constitutive genetic models (where all β-cells have the same genotype), is not clear.

**Table 2:** Genetic mouse models with phenotypes of β-cell senescence and apoptosis.

Genetic model	Senescence markers observed in beta cells	Mouse Phenotypes	Apoptotic beta cells also present?	Effect on other islet cells	Reference
*Pttg1^-/-^*	p21, SA-βgal, gH2A.X, p53,	Early loss of beta cell mass, insulin deficiency, diabetes	Yes	Some alpha cells affected	(99)
*RbΔ7* knock-in	p21, SA-βgal, gH2A.X, p53, SASP	Gradual loss of beta cell mass, insulin deficiency, diabetes	Yes	Not tested	(102)
*Tp53^R172P^; Lig4^-/-^*	p21, p53, SA-βgal, gH2A.X,	Early Loss of beta cell mass, insulin deficiency, diabetes	Yes	No effect on alpha cells	(100)
*Mafa^S64F/+^*	p21, gH2AX, SA-βgal, SASP, Bcl phenotype	Sex-specific beta cell loss in males, impaired insulin secretion, diabetes	No	Not tested	(3)
*Ercc1^d/-^*	p21, p53, p16	Reduced beta cell mass, decreased beta cell function but not diabetic, increased insulin sensitivity, decreased body weight, higher susceptibility to apoptosis	No	Not tested, although alpha cells apparently normal	(101)

In contrast with the above models that lead to a heterogeneous population of senescent and apoptotic β-cells, there are models that show predominantly one or the other. A recent study showed that point mutations in *Rb* that abrogate its phosphorylation sites (and hence prevent cell cycle progression), inhibit post-natal expansion of β-cell mass, resulting in early onset senescence and insufficient β-cell mass to maintain glucose homeostasis (102). A further example is illustrated with MafA*^S64F/+^* mutant mice, in which males exhibit diabetes involving the accumulation of senescent β-cells, whereas females are protected from this phenotype (3), suggesting discrete functional β-cell populations. On the other hand, deletion of the transcription factor *Yy1* leads to profound accumulation of β-cell DNA damage and extreme loss of β-cell mass by apoptosis by 5 weeks of age, without evidence of senescent β-cell accumulation (104). A recently published mouse model might be useful to further understand cellular fate after DNA damage. In the Inducible Changes to the Epigenome (ICE) mice, the endonuclease I-PpoI is used to create double stranded breakages in cells without causing mutations, allowing induction of non-mutagenic cuts *in vivo* (105). Interestingly, during and immediately after I-PpoI induction, there were no detectable changes in cell-cycle profile, apoptosis, or senescence. However, as the mice age, their tissues accumulated senescent cells at a faster rate than control mice (105), suggesting that DNA damage in non-coding regions might preferentially drive senescence versus apoptosis.

Together these genetic models reveal that senescence is finely balanced with apoptosis in β-cells and suggest that the fate choice between these different responses may be dependent on a variety of factors, including not only the type and extent of the damage, but also the age/maturation stage, sex and genetic background.

## 5 Sex differences in β-cell senescence

Sexual dimorphism in disease risk and manifestation has been described across metabolic tissues regulating glucose homeostasis, such as adipose (106), liver (107), and pancreas (108–110). While multiple factors can contribute to (and confound) intrinsic sex differences in diabetes such as age (in relation to puberty and menopause), sex hormone status, ethnicity, obesity, and diet, clinical reports have shown that adult premenopausal women have lower fasting glucose levels and higher insulin sensitivity than men (111, 112). In addition, in those exhibiting prediabetes (defined as HbA1C 5.7-6.4), women tend to manifest with glucose intolerance while men show impaired fasting glucose (reviewed in 113). In general, men are reported to have increased risk of T2D and ketosis-prone diabetes compared to premenopausal women (114), and reports of equivocal rates of T1D in men and women are paradoxical given female susceptibility to autoimmune conditions. Sex differences are also evident at the level of the human pancreatic islet, with women having increased insulin secretion after a glucose load, enhanced GSIS from isolated islets, and increased basal β-cell mass (reviewed in (109). Recent work notes sex differences in not only islet gene expression (115) but also magnitude of transcriptional responses (110). In whole, these reports support intrinsic differences in glucose metabolism and risk for diabetes between men and women.

Biologic sex differences are attributed to a complex interplay of sex chromosomes, physiological sex hormone production, sex hormone receptor status and environmental exposures (including endocrine disruptors) (116). Interpretations from studies using animal and culture models used are often limited by exposure to non-physiologic doses of sex hormone treatment and sex chromosome aneuploidy with progressive passages in cultured cells. Historically, animal research in metabolism were largely performed on male subjects (117). Recent efforts to incorporate sex as a biological variable (SABV) have facilitated the discovery of sex-dependent differences in β-cell function. For example, female mouse islets demonstrate improved resilience to ER stress, with larger capacity for protein production and folding than male islets (110). In line with this, many mouse models of diabetes show a relative protection from disease by female sex, including HFD-fed mice, insulin receptor antagonist (S961) treatment, STZ toxin treatment, and transgenic mice overexpressing hIAPP (reviewed in (109); a notable exception is the NOD mouse model, which manifests a T1D at a higher penetrance in female mice as compared with males (118, 119).

Aging itself is a predominant risk factor in diabetes, and declining levels of sex hormones is tightly linked to advanced tissue aging in natural andropause and menopause in men and women, respectively. Estradiol 17β (E2) demonstrated protective effects from DNA damage, by promoting telomerase expression in skin (120) and reducing p21^Cip1^ and senescence in a breast cancer cell line (121), while testosterone ameliorated vascular senescence (122) and DNA-damage induced senescence in cardiomyocytes (123). In fact, both exogenous E2 and testosterone treatment have been shown to improve β-cell function (124, 125). Chromosomal sex also modifies the senescence program triggered by the DNA damage-inducing agent bleomycin in human donor islets ex vivo. While isolated human islets of both sexes showed increases in p21^Cip1^, the extent of p21^Cip1^ induction varied dramatically (1.5-fold in a male donor versus 12-fold in a female donor) (34, 35), and male islets also showed more robust and sustained activation of DNA damage sensor ATM (34). Further studies addressing sex differences in β-cell senescent signatures and the magnitude of these changes in more human islet donors is warranted.

In several mouse models of diabetes involving β-cell senescence, the metabolic phenotypes show marked sex differences (1, 2), however it is unclear whether the prevalence of senescent β-cells is primarily a function of dysglycemia or biologic sex. The most dramatic example of sexual dimorphism of β-cell function and cellular senescence is in human carriers of a pathogenic variant in the transcription factor MAFA (serine to phenylalanine at position 64, or MAFA^S64F^) (3, 126). This variant promotes MAFA protein stability and predisposes subjects in their late 30’s to either adult-onset diabetes or insulinomatosis (i.e. non-syndromic insulin-producing β-cell tumors). MAFA^S64F^-associated diabetes was much more prevalent (3:1) in males while females presented more often with insulinomatosis (4:1) (126). A mouse model harboring this point mutation in the endogenous *Mafa* locus showed that islet β-cell dysfunction is principally in males, mimicking the findings in human subjects. Significantly, dysfunction in male mice was associated with premature cellular aging and senescence, whereas females had improved glucose clearance without accelerated senescence. In sum, an interplay between risk for β-cell dysfunction, cellular senescence, and biologic sex is likely.

## 6 Translation of therapies targeting β-cell senescence

A long-term goal is to move senescence-targeting therapies towards the clinic as tools to treat and prevent diabetes (56, 57). Preclinical studies in mouse models have validated the use of both genetic and pharmacologic approaches for either targeted removal of proinflammatory senescent β-cells or mitigation of their proinflammatory SASP. Given that pharmacologic approaches targeting senescence are currently undergoing clinical trials, their long term safety and efficacy in other chronic diseases remains to be determined (27, 127). However, it is likely that with further refinement, such approaches could also be tested in trials to improve clinical outcomes for those with or at risk for developing different forms of diabetes. Here we review the literature of preclinical studies targeting β-cell senescence.

### 6.1 Genetic approaches for mitigating β-cell senescence

In the context of T2D, senescent β-cells with SASP can be genetically targeted in mouse models of insulin resistance and HFD using the *INK-ATTAC* transgene. This model incorporates the promoter region of p16^Ink4a^ to drive expression of a drug-activated apoptosis gene (FKBP8-Caspase8) selective in p16^Ink4a^-expressing senescent cells (6) and has been widely used as an *in vivo* reporter and genetic tool to deplete p16^Ink4a^-expressing senescent cells. Upon drug-mediated dimerization, the reporter selectively triggers Caspase8-mediated apoptosis in p16^Ink4a^-expressing cells. Removal of senescent cells using this mouse model on adult HFD mice (7-8 months) or mice with insulin resistance induced by S961 treatment, mitigates glucose intolerance, restores insulin sensitivity concomitant with a reduction in the markers of proinflammatory senescent β-cells in mouse islets (2). More recent work on this model indicates that although it leads to ablation of p16^Ink4a^-expressing senescent β-cells, overall β-cell mass and replication are not affected (96). Similarly, populations of p21^Cip1^-expressing senescent β-cells with SASP are were not affected by genetic deletion of p16^Ink4a^-expressing senescent β-cells (96). Whereas genetic tools have recently been developed to target p21^Cip1^-expressing senescent cells in mice (94, 128), it will be of great interest to apply these to interrogate the roles of p21^High^ senescent β-cells in various diabetes models.

### 6.2 Pharmacologic approaches for mitigating β-cell senescence

Several different pharmacologic approaches have been employed to selectively mitigate the accumulation or phenotypes of senescent β-cells in diabetes models, including senolytics and senomorphics (56, 57). While senolytics are agents that preferentially disarm the anti-apoptosis pathways in senescent cells towards their demise, senomorphics are agents that can modify the biological properties of senescent cells in favor of mitigating their deleterious effects, such as inhibiting/altering the SASP. Several senolytic compounds have been tested *in vivo* to clear senescent cells [reviewed in (129)], such as FOXO4 inhibiting peptide that disrupts binding to p53 (130), the broad spectrum senolytics cardiac glycosides (131), finestin (132) and Heat-shock protein 90 inhibitors (133). More recently, FOXO-DRI (a selective FOXO4 inhibitor) also showed senolytic properties in senescent Leydig testicular cells (134) and in non-small cell lung cancer (135).

Senescent β-cells in different mouse models of diabetes upregulate different anti-apoptotic programs, and thus are sensitive to different types of senolytic agents. For instance, senescent β-cells that accumulate in NOD mice preferentially upregulate Bcl-2 and become sensitive to Bcl-2 family senolytic agents, such as ABT-737 or ABT-199 (1). SA-βgal^+^ cells from 7-8 month old C57BL6 male mice, also are sensitive to ABT-263 (Navitoclax), leading to improved β-cell function and identity (2). Dasatinib + Quercetin (D+Q), a potent combination with broad senolytic activity towards cells using the PI3K/Akt pathway, also elicits senolysis in senescent β-cells of HFD mice (2). Bromodomain ExtraTerminal (BET) domain inhibitors such as JQ1 or iBET-762 act as senomorphics on senescent β-cells in NOD mice by inhibiting these epigenetic readers and thus blocking the SASP at the transcriptional level (35). Administration of endogenously occurring lipids known as palmitic acid hydroxy stearic acids (PAHSAs) can prevent or reverse early stages of β-cell senescence in NOD mice by interfering with the Mdm2/p53 pathway (32).

Sex dimorphism in β-cell senescence responses can impact outcomes to senolytic therapy. Senolytic agents either targeting BCL2 family members or treating with PAHSAs, in female NOD mice improved β-cell function and delayed and/or prevent diabetes progression (1, 32). Notably, only NOD females were used in these studies due to their higher penetrance for diabetes. Senescent β-cells produced in male mice after acute exposure to insulin receptor antagonist S961 were cleared with ABT-263 coincident with improved glycemic responses (2). Similarly, S961-treated males treated with the senolytic cocktail D+Q also improved glucose tolerance and reduced rates of SA-βgal^+^ cells in the final four days of treatment (2). HFD treatment also showed overt diabetes with increases in senescence/aging indices in males and females. In contrast to HFD-treated males, HFD-treated females treated with ABT263 did not show an improvement in glucose tolerance despite reductions in SA-βgal^+^ β-cell population, and Aging and SASP indices, suggesting sex differences in response to senolytics depends on the metabolic disturbance provoking dysglycemia (2). Intriguingly, males and female human islets from non-diabetic donors or those with T2D treated with ABT263 similarly showed increased clearance of senescent β-cells.

However, a significant drawback to the use of these systemically administered agents (senolytics, senomorphics) is the possibility of off-target effects: while senescent cells are preferentially sensitive to these compounds, the drug targets are also expressed in healthy β-cells as well as other cell types. Current efforts are being made to preferentially target senescent cells in a tissue specific manner. Along this vein, preventative senolytic/senomorphic therapy for diabetes not only will deplete senescent β-cells but will be safe to use in patients that ultimately would not develop diabetes (57). For example, senolytic therapy in the NOD model have been focused on female mice with evidence of dysglycemia, however full metabolic profiling of euglycemic females and males receiving similar preventative treatment have not been assessed. Wild-type euglycemic mice pretreated with the senolytic compounds Fisetin (F) or D+Q showed sexually dimorphic glycemic responses (136). In both treatment groups, adult male mice over time showed mild, but not statistically significant, improvement in glucose tolerance to an i.p. glucose load, while female mice did not show any impact. Notably, while adult mice treated with D+Q had minimal effects in males, females showed increased SASP expression and accumulation of white adipose tissue depots, which may signal detrimental metabolic effects long term, suggesting that such preventative therapy is not benign in all populations.

## 7 Impact of senescence on β-cell on islet function and microenvironment

Senescence can alter β-cell function in many ways that are modified by genetic background and may exhibit differences between T1D and T2D. For instance, in the NOD mouse model for T1D, depletion of senescent β-cells in euglycemic female mice did not lead to a significant improvement in glucose tolerance or GSIS in isolated islets (1) suggesting that senescent β-cells in this model are not overtly deleterious to islet insulin secretion. Similarly, during DNA damage-induced senescence in human donor islets in culture, the extent of GSIS in static assays is remarkably preserved compared to controls (34). Significant differences were apparent in DNA damage-induced senescence in mouse MIN6 (C57BL6 background) and NIT1 (NOD background) β-cell lines, where senescence led to increased GSIS relative to controls (34). In contrast, DNA damage-induced senescence in the human fetal-derived female EndoC-βH5 cell line impaired GSIS (34). These findings suggest that DNA damage-associated senescence can differentially impact β-cell function depending on maturation stage, genetic background, culture model and species, and islet donor characteristics.

In T2D models of HFD or acute S961 insulin resistance, senescence results in an accelerated aging phenotype in β-cells and dramatically impairs insulin secretion. Senescent β-cells show increased expression of disallowed genes (2), which may contribute to their diminished function. In both T1D and T2D, senescent β-cells develop a SASP and it remains unclear how SASP alters or may interfere with the natural secretory machinery of the β-cell. Although upregulation of some of the age-dependent factors on their own do not drive age-related deterioration of β-cells (40, 74), in the long-term, aging results in lower transcriptional fidelity and worse β-cell function (137, 138). Senescence has major effects on cellular metabolism, with changes in mitochondrial function and reactive oxygen species (14), which have yet to be elucidated in the context of senescent β-cells. Further studies are needed to explore the specific metabolic and functional changes that occur in senescent β-cells using more sophisticated techniques, such as Patch-seq (139).

In addition to the impact of senescence directly on the metabolism and secretory function of β-cells, senescent β-cells could also exert paracrine effects on neighboring islet cells by virtue of SASP. Although this idea has yet to be tested, culture model systems of senescence support this concept. Conditioned media transfer experiments show that SASP factors secreted by β-cells are capable of triggering phenotypic changes in naïve recipient cells (1–3, 35). Moreover, induction of SASP in human islets in culture leads to altered glucagon secretion from α-cells, suggesting that SASP from β-cells can affect α-cell function (36). BET inhibitor iBET-762, which reduces SASP, can partially rescue the glucagon secretion phenotype (36), lending further evidence implicating SASP in impaired α-cell function. Given recent work implicating α-cell dysfunction in T1D (139–141), further investigation into how the accumulation of senescent β-cells in the islet may affect α-cell function is warranted. Since α-cell function is also impaired in T2D (142), it will be of great interest to explore how senescent β-cell accumulation in T2D is related to alterations in glucagon secretion.

## 8 Conclusions and future directions

In conclusion, it has become clear that β-cell senescence contributes to the pathophysiology of several forms of diabetes. Advancing our understanding of this stress response in the β-cell, its molecular control and heterogeneity, and how it is modified by sex, genetic background and maturation stage to impact other cells in the islet microenvironment holds promise for developing new therapies for treating diabetes. This field of investigation is still in its infancy and therefore establishing best-practices for characterizing senescence and developing accurate markers of senescence in β-cells is of utmost importance through collaborative efforts and concerted initiatives, such as the NIH SenNET [SenNet Consortium (143)]. The identification of such biomarkers will allow the translation of senotherapeutics into the clinic in the setting of diabetes. Care should be taken to accurately characterize senescence phenotypes at multiple levels (gene expression, protein and phenotypic) to avoid ambiguity and discriminate senescence from other β-cell stress responses. In addition, there is a need to continue identifying and testing therapeutic agents that target the vulnerabilities of senescent β-cells and neutralize their deleterious effects, while also minimizing the impact of such treatments on healthy β-cells. As the field matures, β-cell senescence will undoubtedly provide fruitful opportunities for advancing basic islet biology in addition to clinical translation efforts for diabetes.

## Author contributions

JC, CA-M, and PT conceived the ideas together and jointly wrote and edited the manuscript. All authors contributed to the article and approved the submitted version.
